# Consumer Exposure to Antimicrobial Resistant Bacteria From Food at Swiss Retail Level

**DOI:** 10.3389/fmicb.2018.00362

**Published:** 2018-03-06

**Authors:** Christoph Jans, Eleonora Sarno, Lucie Collineau, Leo Meile, Katharina D. C. Stärk, Roger Stephan

**Affiliations:** ^1^Laboratory of Food Biotechnology, Institute of Food Nutrition and Health, Department of Health Science and Technology, ETH Zurich, Zurich, Switzerland; ^2^Institute for Food Safety and Hygiene, University of Zurich, Zurich, Switzerland; ^3^SAFOSO AG, Liebefeld, Switzerland

**Keywords:** antimicrobial resistance, antibiotic resistance, retail foods, food safety, exposure assessment, one health, prevalence

## Abstract

**Background:** Antimicrobial resistance (AMR) in bacteria is an increasing health concern. The spread of AMR bacteria (AMRB) between animals and humans via the food chain and the exchange of AMR genes requires holistic approaches for risk mitigation. The AMRB exposure of humans via food is currently only poorly understood leaving an important gap for intervention design.

**Method:** This study aimed to assess AMRB prevalence in retail food and subsequent exposure of Swiss consumers in a systematic literature review of data published between 1996 and 2016 covering the Swiss agriculture sector and relevant imported food.

**Results:** Data from 313 out of 9,473 collected studies were extracted yielding 122,438 food samples and 38,362 bacteria isolates of which 30,092 samples and 8,799 isolates were AMR positive. A median AMRB prevalence of >50% was observed for meat and seafood harboring *Campylobacter, Enterococcus, Salmonella, Escherichia coli, Listeria*, and *Vibrio* spp. and to a lesser prevalence for milk products harboring starter culture bacteria. Gram-negative AMRB featured predominantly AMR against aminoglycosides, cephalosporins, fluoroquinolones, penicillins, sulfonamides, and tetracyclines observed at AMR exposures scores of levels 1 (medium) and 2 (high) for *Campylobacter, Salmonella, E. coli* in meat as well as *Vibrio* and *E. coli* in seafood. Gram-positive AMRB featured AMR against glycoproteins, lincosamides, macrolides and nitrofurans for *Staphylococcus* and *Enterococcus* in meat sources, *Staphylococcus* in seafood as well as *Enterococcus* and technologically important bacteria (incl. starters) in fermented or processed dairy products. Knowledge gaps were identified for AMR prevalence in dairy, plant, fermented meat and novel food products and for the role of specific indicator bacteria (*Staphylococcus, Enterococcus*), starter culture bacteria and their mobile genetic elements in AMR gene transfer.

**Conclusion:** Raw meat, milk, seafood, and certain fermented dairy products featured a medium to high potential of AMR exposure for Gram-negative and Gram-positive foodborne pathogens and indicator bacteria. Food at retail, additional food categories including fermented and novel foods as well as technologically important bacteria and AMR genetics are recommended to be better integrated into systematic One Health AMR surveillance and mitigation strategies to close observed knowledge gaps and enable a comprehensive AMR risk assessment for consumers.

## Introduction

### Rationale

Antimicrobial resistance (AMR) in commensal bacteria as well as opportunistic or obligate pathogens is an increasing public health concern due to the possibility of AMR gene transfer from commensals to pathogens or among pathogens (Friedman, 2015; ICF, 2017). Food plays an important role in the transmission of commensal microorganisms (MO) but also foodborne pathogens including zoonotic organisms. Transmission of MO between food and humans occurs during handling of raw materials as well as cross- and re-contamination between different food products at production, distribution, and household levels (Macdonald et al., 2015).

Transmission of AMR bacteria (AMRB) to humans and to the human gastro intestinal tract is of concern due to either direct infections or the possibility of horizontal gene exchange with other, potentially pathogenic members of the gut microbiota favored by the high cell densities found in the gut (Aarestrup et al., 2008; Haug et al., 2011; Collignon et al., 2016).

Several European countries implemented surveillance programs for AMR such as DANMAP in Denmark, Swedres-Svarm in Sweden or anresis in Switzerland (Danmap, 2014; Federal Office of Public Health Foph Division Communicable Diseases, 2015; Swedres-Svarm, 2015). Surveillance data from individual European countries are further compiled under the umbrella of the European Centre for Disease Control (ECDC) and European Food Safety Authority (EFSA) to prepare comprehensive annual reports on AMR prevalence dynamics in key bacteria groups in animals, food (mainly raw meat) and human medicine (EFSA and ECDC, 2016). The One Health concept (Zinsstag et al., 2011) has become an integrative part of AMR surveillance in order to combine surveillance approaches, reports and interventions in human and veterinary settings. The Canadian Integrated Program for Antimicrobial Resistance (CIPARS) surveillance scheme takes this idea even further and proposes the additional integration of the environment, feed, companion animals, wildlife as well as food and water into their AMR surveillance program (Deckert et al., 2015). However, specific surveillance programs of food at retail level to determine the exposure of the consumer to AMR bacteria (AMRB) via food are rarely systematically implemented and mainly limited to raw meat samples in combination with human and veterinary medicine data (Federal Office of Public Health Foph Division Communicable Diseases, 2015; EFSA and ECDC, 2016). The integration of food at retail level would contribute to the identification of potential transmission pathways of AMRB between animals and humans and enable the further optimization of AMR mitigation strategies in surveillance and response systems under the One Health concept.

### Objectives

The aim of this study was to evaluate the prevalence of AMRB in food at retail level in Switzerland in order to estimate the potential exposure of Swiss consumers to AMRB in food. A systematic literature review was performed. Data on AMRB and associated AMR genes were analyzed to summarize the current state of knowledge, point out significant knowledge gaps, suggest appropriate interventions following the One Health concept and contribute systematic AMR exposure data to the design of an AMR risk assessment for food at retail and the consumer.

### Research question

What is the exposure of a consumer in Switzerland to AMRB from food at retail level?

## Materials and methods

### Study design and search strategy

#### Description of the study area

Switzerland has a traditional agriculture sector comprised of approximately 50 k farming businesses of which over 3/4 operate in the animal production sector. Switzerland accounted for approximately 1.5 million cattle, 1.5 million swine, 350 k sheep, 70 k goats, and 11 million chicken as the major livestock categories in 2015 (Bundesamt für Statistik BFS, 2016). Primary production of cereal, potato, sugar beets, vegetables, fruit, and berry reached a total of approximately 4,200 k tons in 2014. Animal production accounted for 475 k tons of meat, half of which was of pork origin, 4,067 k tons of cow milk and nearly 50 k tons of eggs. Over 1/3 of the milk is further processed to cheese, approximately 1/4 is used for processed dairy products (butter, yogurt, cream, long-life products) and roughly 1/8 each is used for direct milk consumption by humans and as animal feed (Bundesamt für Statistik BFS, 2016). Consumption of animal products per capita per year reached 250 kg of milk and milk products (without butter), 50 kg of meat. Fruits and vegetables accounted for slightly more than 100 kg each (Bundesamt für Statistik BFS, 2016).

Switzerland's population consumed in 2012 approximately 4 × 10^4^ TJoule of energy from food, of which approximately 60% were produced domestically and 40% were imported (Schweizerischer Bauernverband, 2012). The overall self-sustainability rate of Switzerland is estimated between 50 and 60% of the total useable energy from food (Bundesamt für Statistik BFS, 2016). Animal products provided a self-sustainability of nearly 75% while plant-based products provided <50% of the useable energy (Bundesamt für Statistik BFS, 2016). Fish and seafood depended nearly 100% on imported products (Schweizerischer Bauernverband, 2012). Switzerland ranks among the countries with the highest per capita food import quantity worldwide (Schweizerischer Bauernverband, 2012). Thus, Switzerland serves as a good model to estimate the potential exposure of a consumer to a wide variety of products from all geographical areas and production schemes of the globe. Outcomes are thus likely relevant for other European countries.

#### Search strategy

The search strategy was based on recent work commissioned and published by the UK Food Standards Agency (Mateus et al., 2015) with the following modifications. Switzerland was defined as the study area including food of global origin relevant to the 95% import quantity per food category (section Definition of Food Categories).

#### Search term definition

A Boolean search approach was employed to join variables of food categories with country names and country adjectives including different spelling schemes such as United Kingdom, Great Britain, UK, or England. The basic Boolean search term was defined as: (food category OR alternative food category names) AND (antibiotic OR antimicrobial) AND resistance AND (country name OR country adjective) (Supplementary Data Sheet 1). Search engines (e.g., university repositories) not capable of processing Boolean search terms were searched using basic search terms for “antimicrobial resistance” or “antibiotic resistance” (Supplementary Data Sheet 1). The search was performed on title, abstract, and keywords where available as search setting.

#### PICO and search strategy

The research question (section Research Question) was used to define the Population, Intervention or Exposure, Comparator and Outcome (PICO) (Supplementary Data Sheet 1). The PICO guided the definition of the search terms of interest that were used to identify potential eligible studies for the purpose of the systematic review. The search strategy was applied via science database search engines (Supplementary Data Sheet 1), and also included gray literature websites (e.g., national and international surveillance reports), and experts in the domain area to identify potential relevant studies (Supplementary Data Sheet 1).

#### Definition of food categories

A total of 18 food categories were defined to account for different origins of food, environments, animal species, expected microbiota and intended use (e.g., absence or presence of a heating step prior to consumption), these categories were: (1) fish and seafood; (2) meat cuts, ground, pieces, meatballs; (3) meat cured; (4) meat fermented; (5) meat others (mixed species); (6) milk plain, non-fermented; (7) cheese; (8) milk fermented, not cheese; (9) ice cream; (10) plants/vegetables whole, fresh, unfermented; (11) plants/vegetables fermented; (12) herb/spices; (13) insects; (14) probiotics; (15) technologically important bacteria, starter cultures; food not further specified or containing different types were assigned depending on details available to (16) animal origin, non-fermented; (17) animal origin, fermented; and (18) not further specified.

The search terms per category were developed using general food category terms, specific food product names and Latin names in singular and plural (Supplementary Data Sheet 1).

#### Definition of bacteria categories

The bacteria were grouped into three categories, namely foodborne pathogens (e.g., *Campylobacter, Salmonella, Listeria, Yersinia*), indicator bacteria (e.g., *Escherichia coli, Enterococcus, Staphylococcus*) and technologically important bacteria (e.g., *Lactobacillus, Lactococcus*, and other lactic acid bacteria) (Supplementary Data Sheet 1). Studies for which the bacteria identification was insufficient (e.g., only reporting Gram-negative aerobic mesophiles without further specification) were excluded from further analysis.

#### Definition of relevant countries

Studies linked to Switzerland and Swiss retail sector were targeted. This included countries playing a relevant role in view of the import of food into Switzerland. The aim was to cover 95% of the import quantity in tons per food category based on the 2015 Swiss Federal Import Statistics (Swiss Federal Customs Administration, 2015). Countries were ranked from highest to lowest according to the imported tons of food in 2015 into Switzerland. The import tons per country were then expressed as the percentage of the total import tons of the respective food category. Starting from rank one, the cumulative percentage share of the import quantity per category was calculated until a minimum of 95% of that category was covered by the countries included (Supplementary Data Sheet 1).

#### Inclusion/exclusion criteria

**The inclusion criteria were defined as:**

- Studies focusing on food items of selected food categories at retail level (see section Definition of Food Categories); studies performed on carcasses of rabbit or poultry were retained if conducted at retail level; raw food/meat mixtures of different animal species (e.g., bovine and swine) were included in a separate category
- Studies on domestically-produced (CH) and imported foods (non-CH) according to relevance for Swiss market (Supplementary Data Sheet 1)
- Studies, theses and other documents including gray literature matching the inclusion criteria published between January 1, 1996 and May 30, 2016
- Scientific expert opinion reports (e.g., EFSA) deemed relevant to the research questions were considered if published during the time frame of 1996–2016
- Theses completed at the Universities of Zurich, Bern, Basel, Lausanne, and Swiss Federal Institutions of Technology in Lausanne (EPFL) and Zurich (ETH Zurich) during the time frame of 1996–2016
- Full text manuscripts of papers that were published in English, French, German, and Italian

**The exclusion criteria were defined as:**

- Studies focusing on processed foods, particularly by sterilization, irradiation, or ultra-high temperature treatment (UHT)
- Studies on food mixtures of raw and cooked or fermented food if tracking of bacteria of each category could not be facilitated
- Outbreak reports because the origin of the bacteria was no longer directly causal to the retail food
- Studies on animal products and fresh produce at farm or abattoir level such as bulk tank milk or carcasses (with the exception of poultry and rabbits as mentioned above) or farm environment including water in fish aquacultures
- Any type of study (or part of a study) that assessed frequency of resistance, transmission of resistant bacteria or resistance determinants to humans in/from the following sources:
  - Companion animals (including horses) or exotic pets
  - Direct contact with wildlife
  - Healthcare settings (nosocomial infections) unless primary cause was a foodborne pathogen of animal origin (i.e., pork or poultry meat) or from fresh produce (i.e., fruit or vegetables, including fresh salad)
  - Occupational settings in veterinary practice
  - Humans, when humans are deemed to be the source of primary contamination [e.g., methicillin resistant *Staphylococcus aureus* (MRSA) human clones at community level]
- Studies were data was only reported in graphs, figures or histograms where values were not extractable
- Literature regarding mathematical models used to evaluate AMR

### Literature data treatment and curation of reference lists

All search hits were imported into the reference management software (Endnote X7.4, Clarivate Analytics, Philadelphia, PA, USA) to collate the identified literature. All duplicates were removed prior to the first stage sifting process electronically via the reference software and subsequently also manually curated. Subsequently, the list was filtered for positive and negative keywords derived from food categories, food environment and other settings of inclusion or exclusion aspects (detailed list available in Supplementary Data Sheet 1). References featuring only negative keywords were excluded. References featuring only positive keywords were included. References featuring positive and negative keywords were manually checked prior to inclusion or exclusion.

All identified studies and other relevant literature were screened for eligibility using a three-stage sifting approach to review the title, abstract, and full text adopting a single reviewer approach for each study. A random check of excluded studies was conducted by a second reviewer and any discrepancies observed were discussed. The number of documents identified and screened out were recorded at each stage. Reasons for exclusion were recorded.

### Assessment of risk of bias

The risk of bias assessment was conducted on the 1,065 references retrieved for full analysis. Primary evaluation was based on validation of inclusion/exclusion criteria, relevant countries, ability to differentiate sample source, isolate source, and data extraction. Subsequent 313 references were categorized in three categories based on the sampling scheme employed (1) convenient, non-systematic or randomized/probabilistic sampling if no details were provided on how samples were obtained or only stated as random sampling without details on randomization. (2) Systematic approach, not further specified was selected for studies employing a systematic description of their sampling approach but no specification on sample selection or then employed basic convenient sampling. (3) Systematic, weighted, and probabilistic sampling was only selected for studies that fully described all aspects of sample selection. These four categories also represent the range of (1) high potential bias, (2) intermediate potential bias, and (3) low potential bias. Expert opinions and literature reviews were not assessed for bias; nevertheless, findings of these studies were reported as part of the systematic review. Bias was assessed following the criteria stipulated by the PRISMA statement (Liberati et al., 2009).

### Data extraction

Data relating to study type, food categories, country of food production, food purchase, and food analysis, number of samples, isolates, phenotypic, and genotypic AMR as well as laboratory testing methodologies used were extracted into an Excel document (Microsoft Office, Microsoft, Redmond, WA, USA).

### Data analysis

#### Descriptive analysis of all included studies

A descriptive analysis was first performed on all studies conforming with the inclusion criteria, including those based on small sample sizes, in order to be aware of present but not quantifiable risks. Data were further described by food categories, observed phenotypic and genotypic resistances and corresponding bacteria species.

#### Quantitative analysis of a subset of included studies

##### Criteria defined for studies to qualify for quantitative analysis

Next, a quantitative analysis of AMR prevalence and subsequent exposure assessments was conducted on a subset of studies matching refined criteria, namely (1) the number of AMR positive samples (or isolates) and the total number of samples (or isolates) tested were available, and (2) a minimum of 30 samples and 15 isolates were tested. These cutoff values of 30 samples and 15 isolates were used as a compromise between having a sufficient level of precision and power of detection around the bacteria prevalence and AMR prevalence estimate, respectively, having a sufficient number of isolates to subsequently estimate the AMR prevalence and keeping a sufficient number of studies in the analysis.

##### Quantitative analysis of AMR prevalence

Based on the data extracted, the minimum, median, and maximum proportion of AMR samples or isolates was calculated in MS EXCEL across available studies and by combination of food category, food item, bacterium species, and antimicrobial class (AM class). Median AMR prevalence >0.5 was considered as high and a significant risk.

##### Exposure assessment

A qualitative exposure assessment was conducted following the *Codex alimentarius* framework (Food and Agriculture Organisation and World Health Organization, 2011), in order to estimate the level of exposure to food contaminated with selected AMRB at retail. The exposure assessment consisted of a combination of three scores (Supplementary Data Sheet 1): (i) the score of consumption of each selected food item (food consumption score); (ii) the score of food contamination with the bacteria of interest, being antimicrobial resistant or not (bacteria prevalence score); and (iii) the score of AMR observed among food contaminating bacteria (AMR prevalence score), which were then used to calculate the overall AMR prevalence score and AMRB exposure score. Each score ranged from 0 to 2 for lowest to highest values, respectively.

##### Definition of food consumption score

For the food consumption score, food categories were grouped into four main food types of meat, plants, seafood/fish, and dairy products. Meat included pork, poultry, beef, veal, lamb, game, and mixed meat. Plants included fresh fruits and vegetables. Seafood and fish included a diversity of fishes, mollusks, and crustaceans. Dairy products included raw milk and processed dairy products, which summarized powdered milk, hard and soft cheese, and yogurts. Studies for which no distinction could be made between foods from different categories (e.g., meat reported together with dairy products) were excluded from further analysis. For each food item the average level of consumption in Switzerland (in grams/person/day) was estimated using the menuCH survey and the annual report of the Swiss meat industry (Proviande, 2016; Federal Food Safety Veterinary Office, 2017). Based on the observed distribution of the food item consumption levels, a 3-point food consumption score was defined (Supplementary Data Sheet 1).

##### Definition of bacteria prevalence score

For each separate study, the level of food contamination with the bacteria of interest was estimated by dividing the number of samples contaminated with the bacteria (resistant or not) by the total number of food samples that were tested. The bacteria prevalence was estimated for each individual food item as the median bacteria prevalence observed across all studies that investigated the prevalence of the bacteria in the food item. Based on the observed distribution of the bacteria prevalence, a 3-point bacteria prevalence score from 0 to 2 was subsequently defined (Supplementary Data Sheet 1).

##### Definition of AMR prevalence score

The AMR prevalence score was based on the observed AMR profiles, being obtained either from phenotypic or genotypic assays. The observed AMR profiles were grouped into resistance to 24 AM classes. AMR to aminoglycosides, carbapenems, cephalosporins, fluoroquinolones, glycopeptides, macrolides, phenicols, polypeptids, and tetracyclines were particularly highlighted in the exposure assessment as these AM classes are considered to be of major public health relevance (World Health Organization, 2016). For each separate study and each bacteria species of interest, the AMR prevalence to a given AM class was estimated by dividing the number of resistant isolates by the total number of isolates that were tested for AMR. The AMR prevalence against a given AM class was estimated for each individual food item and each bacteria species, as the median AMR prevalence observed across all studies that investigated the AMR prevalence against this AM class for the food item and the bacteria species of interest. Based on the observed distribution of the AMR prevalence, a 3-point AMR prevalence score was subsequently defined (Supplementary Data Sheet 1).

##### Definition of overall AMR prevalence score

In order to get an overview of the overall level of AMR observed for each combination of food item and bacteria species, an overall AMR prevalence score was defined based on the sum of the AMR prevalence scores observed for the nine AM classes of major public health relevance (Supplementary Data Sheet 1). In case the AMR prevalence of a given AM class was not available (i.e., no included study investigated the prevalence of AMR against this particular class and for this particular food item/ bacteria species combination), the associated AMR prevalence score was assumed to be low (AMR prevalence score = 0).

##### Definition of AMRB exposure score

A risk matrix was subsequently used to combine the food consumption, bacteria prevalence and AMR prevalence scores following a 2-step approach. First, the bacteria prevalence score was combined together with the overall AMR prevalence score in order to obtain an AMRB prevalence score (Supplementary Data Sheet 1). Next, the food consumption score was combined together with the AMRB prevalence score in order to obtain an AMRB exposure score (Supplementary Data Sheet 1).

##### Definition of level of evidence of exposure

For each combination of food item and bacteria species, the level of evidence of exposure to the AMRB of interest was described by providing the number of studies from which the AMRB exposure score was estimated. Estimates based on one or two, three to five, and more than five studies were believed to provide low, medium and strong levels of evidence, respectively, assuming each study provided similar levels of evidence.

## Results

### General findings from the descriptive analysis of all included studies

**Figure 1:**
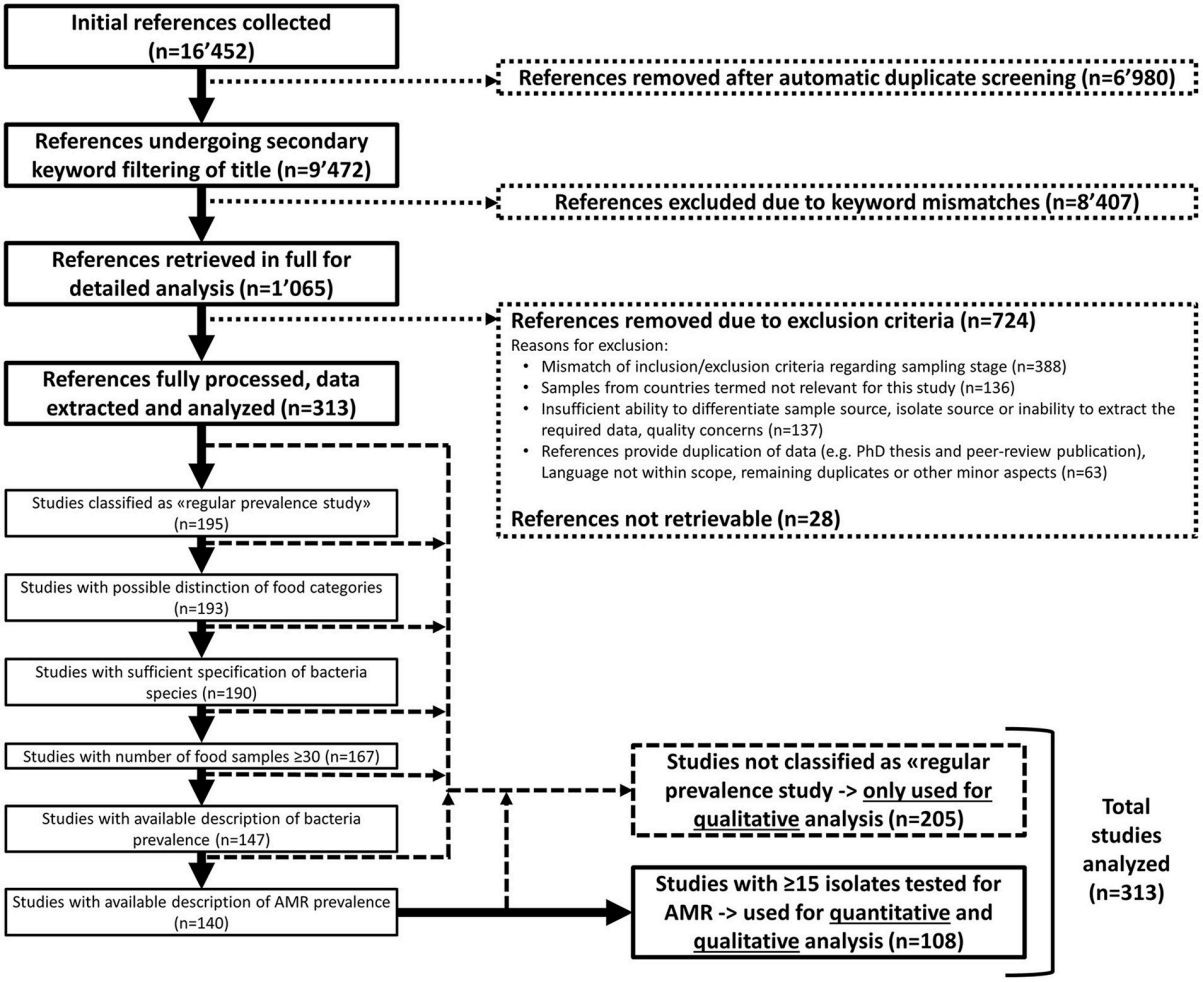
Article selection process for quantitative and qualitative analysis.

The search for AMR literature yielded a total of 16,452 references (Figure 1). Detailed evaluation was performed on 1,065 references, which were further reduced to 313 references by inclusion and exclusion criteria for quantitative or qualitative analysis (Figure 1). The majority of studies (*n* = 388) out of the 1,065 were excluded due to topics that did not match the inclusion criteria e.g., because samples were obtained not at retail level but from abattoirs, farm environments, animals, or humans (Figure 1).

Out of the 313 included studies, the geographic origin was differentiated by place of production, collection and analysis. The 313 studies represented food products from 37 different geographic classifications (Supplementary Data Sheets 1, 4). If a country was not further specified, geographic summary terms such as EU others, South America or various were applied. The data extracted encompassed 122,438 food samples (Table 1). The majority of these samples were collected and analyzed in Europe (30.0%) or North America (59.5%). Europe and North America were also accountable for 36.3 and 57.3% of AMR positive samples, respectively (Table 1). Switzerland featured an AMR positive sample prevalence of 35.7% and AMR positive strain prevalence of 33.0% at collection stage. At production stage, AMR positive sample and AMR positive strain prevalence was determined at 49.1% and 41.5%, respectively (Table 1). Hotspots were observed particularly for *E. coli* (*n* = 9,280 of 12,664), *Salmonella* (*n* = 572 of 2,570) and *Staphylococcus* (*n* = 466 of 1,879) from the USA or *Campylobacter* from Canada (*n* = 3,240 of 6,909) and the UK (*n* = 2,609 of 6,909) (Supplementary Data Sheet 2).

**Table 1:** Overview of included food samples, their geographic origin and proportion of samples from which AMR bacteria were retrieved.

	**Number (%-share of total//%-share within country)^a^**								
	**Total**	**Europe^b^ (incl. Switzerland)**	**Switzerland**	**Asia^c^**	**Africa^d^**	**North America^e^**	**South America^f^**	**Caribbean^g^**	**Global, unspecified country**
Samples collected from food produced in the area	122,438 (100.0)	15,648 (12.8)	670 (0.5)	2,616 (2.1)	2,648 (2.2)	2,961 (2.4)	810 (0.7)	50.0 (0.0)	97,705 (79.8)
AMR positive samples collected from food produced in the area	30,092 (100.0//24.6)	3,423 (11.4//21.9)	329 (1.1//49.1)	550 (1.8//20.6)	133 (0.4//5.0)	738 (2.5//24.9)	168 (0.6//20.7)	11.0 (0.0//22.0)	25,069 (83.3//25.7)
Samples collected in the area	122,438 (100.0)	36,715 (30.0)	2,404 (2.0)	5,392 (4.4)	3,593 (2.9)	72,960 (59.5)	3,778 (3.1)	0 (0.0)	0 (0.0)
AMR positive samples collected in the area	30,092 (100.0//24.6)	10,942 (36.4//29.8)	859 (2.9//35.7)	1,142 (3.8//21.0)	173 (0.6//4.8)	17,239 (57.3//23.6)	596 (2.0//15.8)	0 (0.0)	0 (0.0)
Strains collected from food produced in the area	38,362 (100.0)	7,648 (19.9)	1,009 (2.6)	1,352 (3.5)	929 (2.4)	1,634 (4.3)	432 (1.1)	12 (0.0)	26,355 (68.7)
AMR positive strains collected from food produced in the area	8,799 (100.0//23.0)	2,412 (27.4//31.5)	419 (4.8//41.5)	305 (3.5//22.5)	275 (3.1//29.6)	779 (8.8//47.7)	133 (1.5//30.8)	1 (0.0//8.3)	4,894 (55.6//18.6)
Strains collected in the area	38,362 (100.0)	16,173 (42.1)	2,064 (5.4)	2,098 (5.5)	951 (2.5)	17,720 (46.2)	1,306 (3.4)	0 (0.0)	114 (0.3)
AMR positive strains collected in the area	8,799 (100.0//23.0)	4,532 (51.4//28.0)	682 (7.8//33.0)	591 (6.7//28.1)	278 (3.2//29.2)	2,864 (32.5//16.2)	498 (5.7//38.1)	0 (0//0)	36 (0.4//31.6)

a *%-share of total: calculated in relation to the total number of samples/strains in that row; %-share within country: calculated only for AMR positive samples or strains in relation to the number of samples/strains obtained within that same geographic origin*.

b *Austria, Belgium, Czech Republic, Denmark, France, Germany, Greece, Hungary, Ireland, Italy, Netherlands, Northern Ireland, Poland, Portugal, Slovenia, Spain, Sweden, Switzerland, UK*.

c *Bangladesh, China, India, Indonesia, Japan, Thailand, Turkey, Vietnam*.

d *Egypt, South Africa*.

e *Canada, Mexico, USA*.

f *Argentina, Brazil, South America (not specified)*.

g *Dominican Republic*.

**Figure 2:**
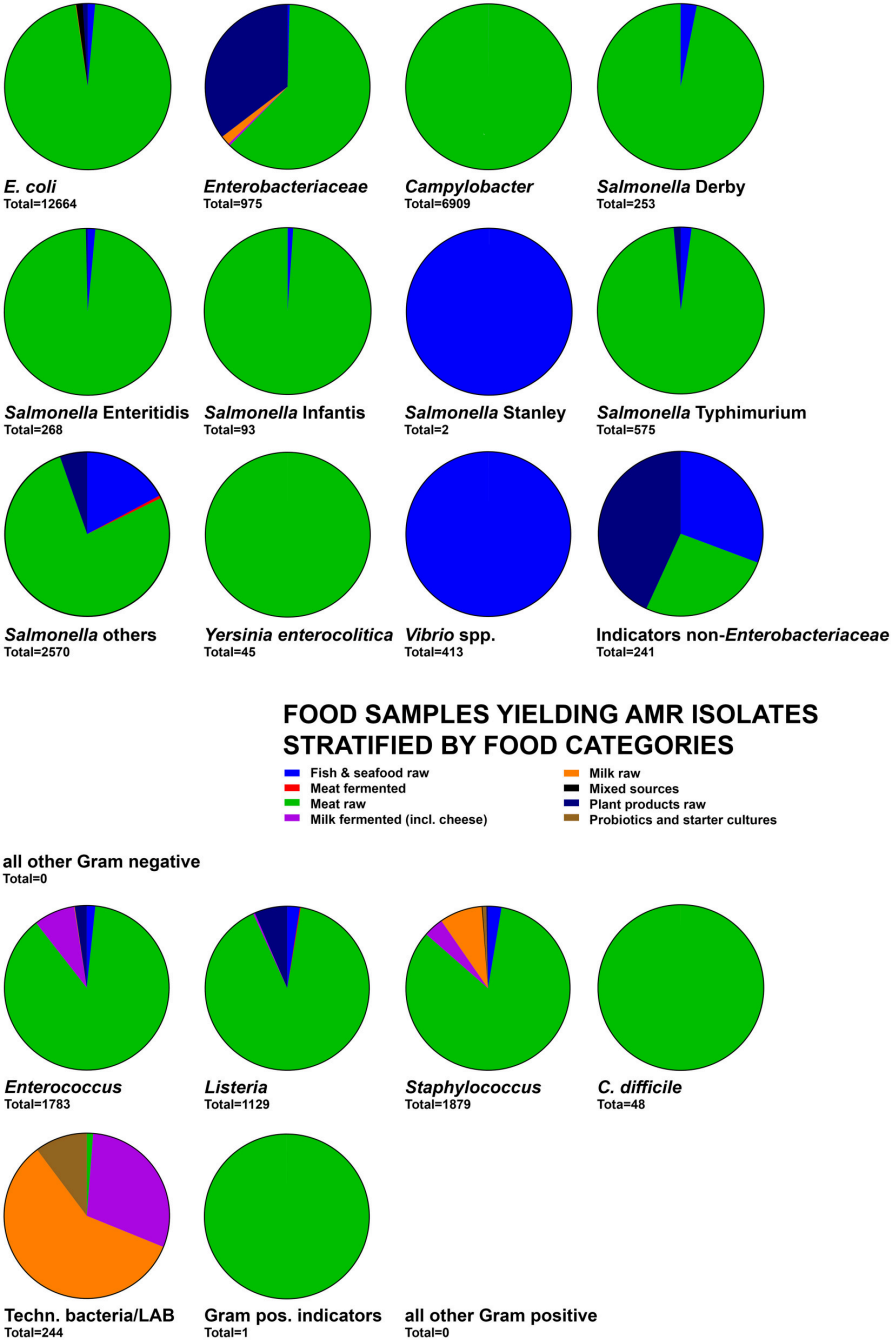
Distribution of the number of food samples detected positive for AMR isolates and stratified by food categories. The total below each pie chart indicates the total of samples used to calculate the graph. For other Gram-negative, other Gram-positive, and Gram-positive indicator groups, no or only limited quantitative sample association was possible and thus no diagram was obtained despite the presence of AMR phenotypes in these groups.

**Figure 3:**
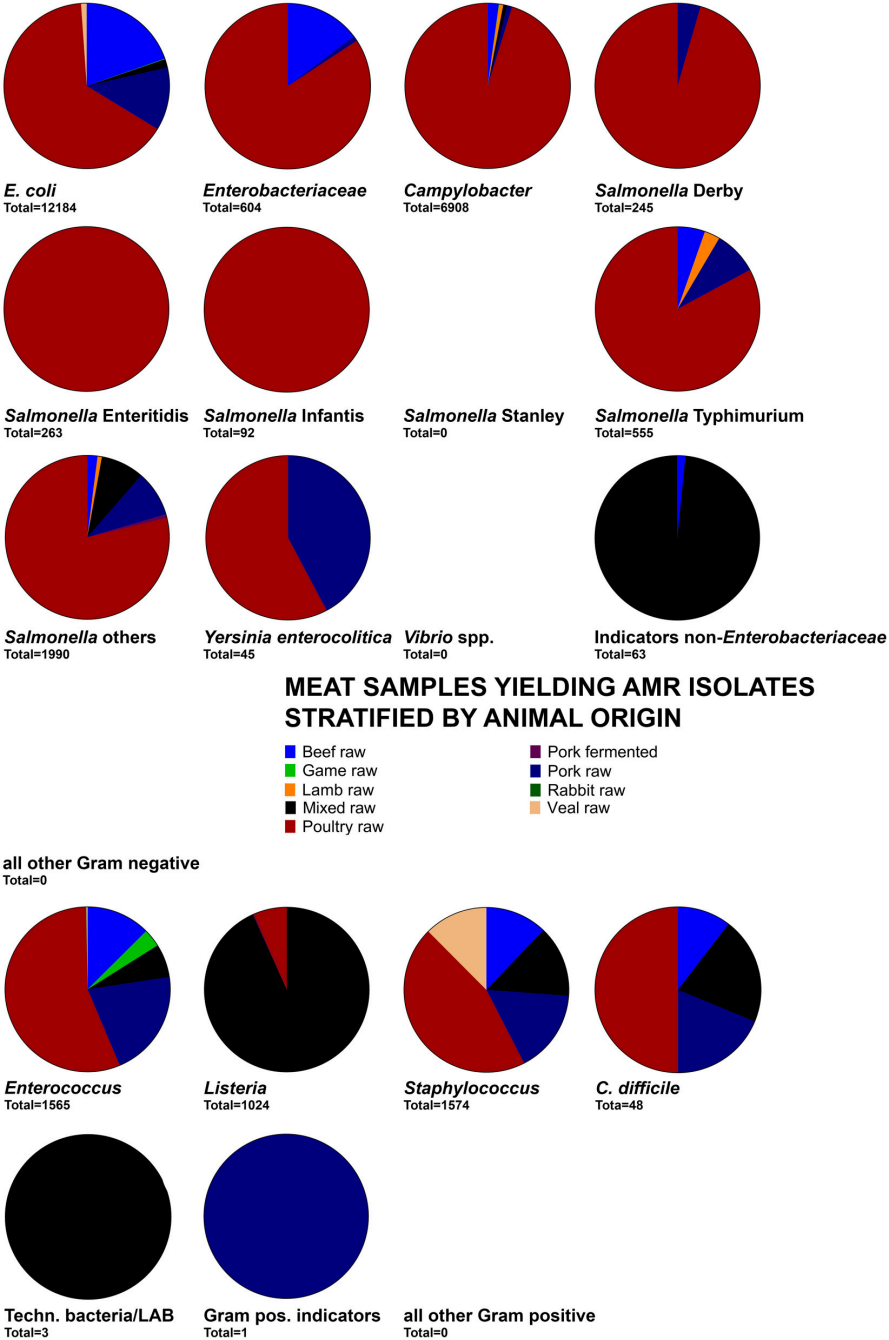
Distribution of the number of meat samples yielding AMR isolates by food categories. The total below each pie chart indicates the total of samples used to calculate the graph.

The food origin for the majority of Gram-negative and Gram-positive foodborne pathogens as well as indicator bacteria related to meat products (Figure 2) and particularly raw poultry meat (Figure 3). Exceptions relating to fish and seafood products were associated with *Salmonella* Stanley and *Vibrio* spp. whereas milk products were mainly a source for AMR *Enterococcus, Staphylococcus*, and technologically important bacteria (Figure 2). The large abundance of meat-derived AMRB was also related to an overrepresentation of studies on raw meat (*n* = 160) and raw fish or seafood products (*n* = 54) among the 313 studies included (Table 2). Out of these studies, the total of 122,438 food samples and 38,362 bacteria yielded 30,092 (24.6%) samples and 8,799 (22.9%) bacteria positive for AMR (Table 3). The majority of samples (61.5%) and bacteria (70.2%) originated from meat products (cuts, ground pieces, or meatballs) and were also responsible for the majority of detected total AMR positive samples (88.7%) and bacteria (60.1%) (Table 3). Relative AMR abundance was therefore calculated to account for the overrepresentation of raw meat and seafood samples in the overall sample set. The highest relative abundance of AMR positive samples among categories featuring >100 samples was observed in meat products 32.6–35.5% followed by cheese at 26.2% (Table 3). For categories with 10–20 samples, fermented dairy products, and ice cream featured highest AMR positive prevalence at 27.8 and 50.0%, respectively (Table 3). Generally, relative AMRB prevalence was high among fermented products of all types ranging from 31.8% in fermented meat to 61.3% in fermented plant products (Table 3). Due to the inability to deduce sample and strain origins among probiotics and starters, detected relative AMR prevalence could not be quantified (Table 3). Focusing on samples and bacteria obtained of Swiss retail origin yielded a similar outcome. Out of 2,404 samples, 859 (35.7%) were positive for AMRB. Furthermore, 682 out of 2,064 isolates (33.0%) were AMR bacteria (Table 4). Highest AMRB prevalence was observed in meat products (cuts, ground pieces, or meatballs) at 37.6% followed by other meat products (27.6%) and cheese (19.1%) (Table 4). Interestingly, 13 out of 18 (72.2%) tested starter cultures harbored AMRB yielding 10 out of 23 AMRB isolates (43.5%) (Table 4).

**Table 2:** Overview of food product categories address by the studies included for qualitative and quantitative analysis^a^.

**Raw/fresh**				**Cured meat**	**Fermented**			**Other**				
**Fish/seafood**	**Meat**	**Milk**	**Plants**		**Meat**	**Milk**	**Plants**	**Insect**	**Probiotic/starter**	**General food^b^, animal origin**	**General food^b^, not further specified**	**Mixed food**
54	160	22	39	5	16	36	4	1	5	8	3	4

a *The total does not equal n = 313 as several studies reported on multiple product categories and were thus counted once for each category*.

b *General food: this term was used for food items where raw or fermented status could not be extracted*.

**Table 3:** Overview of samples of global origin stratified by food category from which AMR bacteria were retrieved.

	**Number (%-share of total number//%-share within food category)**																		
	**Total**	**Fish and seafood**	**Meat**				**Milk**				**Plant**			**Other**					
			**Cuts, ground, pieces, meatballs**	**Cured**	**Fermented**	**Other meat^a^**	**Plain, non-fermented**	**Cheese**	**Fermented, not cheese**	**Ice cream**	**Whole, fresh, unfermented**	**Fermented**	**Herb spices**	**Insects**	**Probiotics**	**Starter**	**Animal origin, non-fermented**	**Animal origin, fermented**	**Not further specified**
Total number of tested samples	122,438 (100.0)	5,665 (4.6)	75,297 (61.5)	386 (0.3)	661 (0.5)	1,321 (1.1)	3,588 (2.9)	1,124 (0.9)	18 (0.0)	40 (0.0)	32,165 (26.2)	20 (0.0)	5 (0.0)	11 (0.0)	2 (0.0)	19 (0.0)	155 (0.1)	12 (0)	1,949 (1.6)
Total number of AMR positive samples	30,092 (100.0//24.6)	1,251 (4.2//21.9)	26,704 (88.7//35.5)	14 (0.0//3.6)	16 (0.1//2.4)	430 (1.4//32.6)	314 (1.0//8.8)	294 (1.0//26.2)	5 (0.0//27.8)	20 (0.1//50.0)	837 (2.8//2.6)	0 (0//0)	2 (0.0//40.0)	0 (0//0)	4^b^ (0.0//200.0^b^)	35^b^ (0.1//184.2^b^)	0 (0//0)	0 (0//0)	166 (0.6//8.5)
Total number of isolates tested	38,362 (100.0)	2,454 (6.4)	26,954 (70.2)	155 (0.4)	899 (2.3)	884 (2.3)	1,212 (3.2)	1,339 (3.5)	55 (0.1)	0 (0.0)	2,326 (6.1)	194 (0.5)	7 (0.0)	0 (0)	4 (0.0)	75 (0.2)	826 (2.2)	8 (0)	970 (2.5)
Total number of AMR positive isolates	8,799 (100.0//22.9)	828 (9.4//33.7)	5,291 (60.1//19.6)	44 (0.5//28.4)	286 (3.2//31.8)	419 (4.8//47.4)	410 (4.7//33.8)	540 (6.1//40.3)	23 (0.3//41.8)	0 (0.0)	548 (6.2//23.6)	119 (1.4//61.3)	7 (0.1//100.0)	0 (0.0)	4 (0.0//100.0)	53 (0.6//70.7)	111 (1.3//13.4)	4 (0//50.0)	106 (1.2//10.9)

a *Other meat: level of processing not described*.

b *Under special circumstances, numbers of AMR positive samples can be higher than the initial number of samples. This is due to the data extraction process and subsequent inability to differentiate between exact isolate origin if a study investigated different species. A sample is then counted as AMR positive once for each species tested positive*.

**Table 4:** Overview of samples of Swiss retail origin stratified by food category from which AMR bacteria were retrieved.

	**Number (%-share of total number//%-share within food category)**																		
	**Total**	**Fish and seafood**	**Meat**				**Milk**				**Plant**			**Other**					
			**Cuts, ground, pieces, meatballs**	**Cured**	**Fermented**	**Other meat^a^**	**Plain, non-fermented**	**Cheese**	**Fermented, not cheese**	**Ice cream**	**Whole, fresh, unfermented**	**Fermented**	**Herb spices**	**Insects**	**Probiotics**	**Starter**	**Animal origin, non-fermented**	**Animal, fermented**	**Not further specified**
Total number of tested samples	2,404 (100.0)	44 (1.8)	1,798 (74.8)	0 (0)	50 (2.1)	29 (1.2)	0 (0)	115 (4.8)	5 (0.2)	0 (0)	340 (14.1)	0 (0)	4 (0.2)	0 (0)	1 (0)	18 (0.7)	0 (0)	0 (0)	0 (0)
Total number of AMR positive samples	859 (100.0//35.7)	66^b^ (7.7//150.0^b^)	676 (78.7//37.6)	0 (0//0)	0 (0//0)	8 (0.9//27.6)	0 (0//0)	22 (2.6//19.1)	5 (0.6//100.0)	0 (0//0)	65 (7.6//19.1)	0 (0//0)	1 (0.1//25.0)	0 (0//0)	3^b^ (0.3//300.0^b^)	13 (1.5//72.2)	0 (0//0)	0 (0//0)	0 (0//0)
Total number of strains tested	2,064 (100.0)	150 (7.3)	1,062 (51.5)	0 (0)	116 (5.6)	7 (0.3)	34 (1.6)	267 (12.9)	5 (0.2)	0 (0)	130 (6.3)	0 (0)	1 (0)	0 (0)	3 (0.1)	23 (1.1)	0 (0)	0 (0)	266 (12.9)
Total number of AMR positive strains	682 (100.0//33.0)	5 (0.7//3.3)	463 (67.9//43.6)	0 (0//0)	16 (2.3//13.8)	2 (0.3//28.6)	2 (0.3//5.9)	148 (21.7//55.4)	5 (0.7//100.0)	0 (0//0)	23 (3.4//17.7)	0 (0//0)	1 (0.1//100.0)	0 (0//0)	3 (0.4//100.0)	10 (1.5//43.5)	0 (0//0)	0 (0//0)	4 (0.6//1.5)

a *Other meat: level of processing not described*.

b *Under special circumstances, numbers of AMR positive samples can be higher than the initial number of samples. This is due to the data extraction process and subsequent inability to differentiate between exact isolate origin if a study investigated different species. A sample is then counted as AMR positive once for each species tested positive*.

### Detected phenotypic and genotypic AMR by key bacteria groups and their respective food product categories

**Figure 4:**
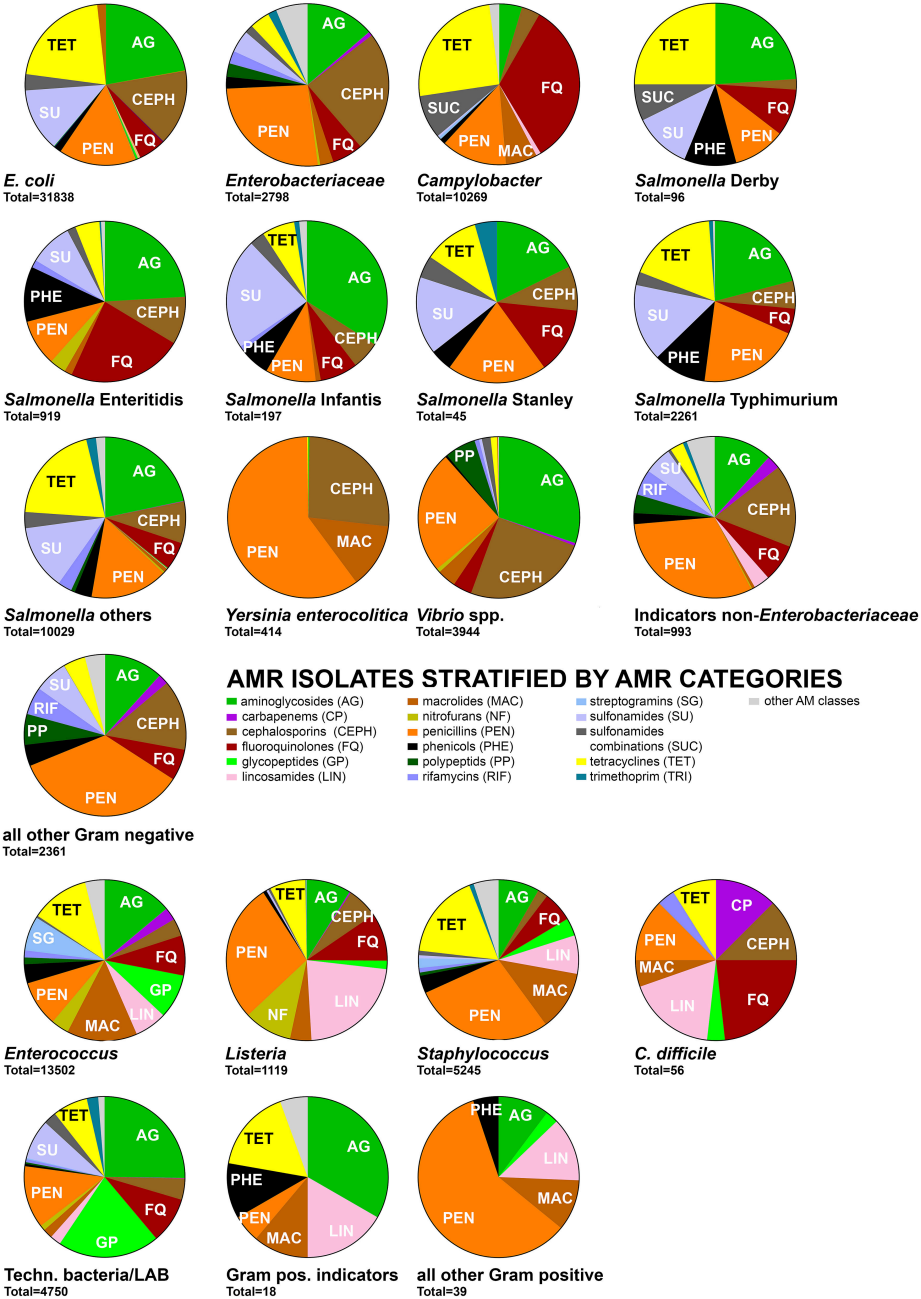
Distribution of the number of AMR isolates of foodborne pathogens, indicator bacteria and technologically important bacteria by detected phenotypic AMR against 17 main AM classes. AM classes contributing ≥5% of AMR isolates per bacteria group are indicated in the pie chart. Data on AMR isolates for further AM classes are summarized under “other AM classes” and available in full in Supplementary Data Sheet 2. An isolate was counted once for each AMR determined.

AMRB were stratified by the defined categories into foodborne pathogens, indicator bacteria, and technologically important bacteria. For further insights, the key species among foodborne pathogens and indicator bacteria were separately evaluated regarding the distribution of AMR phenotypes (Figure 4). The key phenotypic AMR category throughout all groups was penicillin resistance (Figure 4) followed by a high prevalence of aminoglycoside and tetracycline resistances. Cephalosporin and fluoroquinolone resistances were particularly observed among Gram-negative bacteria whereas Gram-positive bacteria featured more frequently glycopeptide and macrolide resistances (Figure 4).

#### Gram-negative bacteria

##### Campylobacter spp.

*Campylobacter coli* and *Campylobacter jejuni* were described in 30 studies analyzing food produced or collected in Austria, Belgium, Canada, Denmark, Germany, Ireland, Portugal, Spain, Switzerland, the UK, and the USA. The major associated food categories were meat of beef, lamb, pork, and poultry as well as fish in raw condition, in pieces, cuts, ground, or frozen form. AMR was observed against various AM, particularly tetracyclines, fluoroquinolones, and penicillins (Figure 4). AMR genotypes were related to genetic mutations in *aphA3, gyrA*, 23S rRNA gene, or *cmeABC* resulting in AMR to aminoglycosides, fluoroquinolones, macrolides, or general multidrug efflux pumps, respectively (Supplementary Data Sheet 2, Qualitative analysis).

##### Escherichia coli

*Escherichia coli* was described in 65 studies. The majority of products were unprocessed animal products including meat, milk, and fish, but also cheese, vegetables and fruits served as reservoir for AMR *E. coli*. Samples originated from North and South America, multiple European countries including Switzerland and few Asian and African countries. AMR was observed at comparable prevalence to aminoglycosides, cephalosporins, penicillins, sulfonamides, and tetracyclines (Figure 4). Genotypic information was well documented and based particularly on variants of *bla*_CMY_, *bla*_CTX_, *bla*_OXA_ or *bla*_TEM_ as well as variants of *drfA, strA/B* and *sul1/2/3* among many others (Supplementary Data Sheet 2, Qualitative Analysis).

##### Salmonella

*Salmonella enterica* subsp. *enterica* was classified according to the five serovars Derby, Enteritidis, Infantis, Stanley, and Typhimurium. All other serovars were grouped together. While all serovars shared a close link to animal products, fish or seafood and to some extent also plant and fruit products, serovar Stanley was exclusively isolated from fish and seafood. Most products with the exception of fermented pork sausages were minimally processed such as cut, pealed, shredded, or minced. A common source for serovars Derby, Enteritidis, Infantis, and Stanley were products originating from and being sold in China, Southeast Asia and southern Europe. *S*. Typhimurium and all remaining serovars were in addition also isolated from products produced in European and North American countries and available in a wider variety of countries including Switzerland. AMR was serovar specific but all shared a high prevalence of aminoglycoside, cephalosporine, fluoroquinolone, penicillin, phenicols, and sulfonamide resistances (Figure 4). Genetic elements encoding for AMR were detected as *bla*_CMY_, *bla*_CTX_, *bla*_OXA_, and *bla*_TEM_ variants as well as *aadA, aphA, dfrA, catA, sat, str, sul1/2/3*, and *tet*A/B/C/G variants (Supplementary Data Sheet 2, Qualitative Analysis).

##### Yersinia enterocolitica

*Yersinia enterocolitica* was exclusively isolated from raw meat, particularly poultry and pork meat (Figures 2, 3). Production areas of food were not reported. Collection areas encompassed Austria, Italy, and Switzerland. Phenotypic AMR was in 2/3 related to penicillins while the remaining 1/3 was related to cephalosporins and macrolides (Figure 4). No AMR genes were reported (Supplementary Data Sheet 2, Qualitative Analysis).

##### Vibrio spp.

The genus *Vibrio* was represented by *Vibrio alginolyticus, Vibrio cholerae, Vibrio litoralis, Vibrio parahaemolyticus, Vibrio vulnificus* and not further specified *Vibrio* spp. It was exclusively isolated from raw or frozen fish and seafood products produced in Europe and Asia. The products were collected and analyzed in various European and Asian countries as well as the USA. Predominant AMRs were related to aminoglycosides, cephalosporins, and penicillins (Figure 4). AMR genes were not reported (Supplementary Data Sheet 2, Qualitative Analysis).

##### Enterobacteriaceae

The family Enterobacteriaceae was represented by *Citrobacter, Cronobacter, Enterobacter, Klebsiella, Kluyvera, Pantoea*, and *Shigella E. coli* and *Salmonella* were grouped as own categories. Enterobacteriaceae were isolated from most types of milk, cheese, meat, plant and seafood products with a predominance of raw meat origin, particularly of beef, and poultry origin (Figures 2, 3). Products were produced, collected and analyzed from most areas worldwide including various countries from European, Asian, and the Americas (Supplementary Data Sheet 2, Qualitative Analysis). Phenotypic AMR was diverse but predominated by AMR to aminoglycosides, cephalosporins and penicillins (Figure 4). The genetic background was related to a large array of *bla*_ACC_, *bla*_CMY_, *bla*_CTX_, *bla*_DHA_, *bla*_FOX_, *bla*_OXA_, *bla*_OXY_, *bla*_SHV_, or *bla*_TEM_ variants. Furthermore, *aac, dfrA* or the concerning colistin resistance encoding *mcr-1* gene were observed (Supplementary Data Sheet 2, Qualitative Analysis).

##### Indicator bacteria non-enterobacteriaceae

This group was comprised of *Acinetobacter, Aeromonas, Pseudoalteromonas*, and *Pseudomonas*. They were predominantly isolated from meat, milk, fish and plant products, particularly raw products and also some cheeses produced and collected mainly in European countries including Switzerland (Figures 2, 3, Supplementary Data Sheet 2, Qualitative Analysis). Phenotypic AMR was detected predominantly against aminoglycosides, cephalosporins and penicillins, besides AMR against fluoroquinolones, macrolides, phenicols, polypeptids, and rifamycins at lower prevalence (Figure 4). Knowledge on genetic AMR background was limited to *bla*_CTX−M−14_ (Supplementary Data Sheet 2, Qualitative Analysis).

#### Gram-positive bacteria

##### Clostridium difficile

*Clostridium difficile* affected only meat products as well as seafood and fish in raw, cut, whole, or frozen forms. Production origins were across Europe including Switzerland as well as Canada and the USA. AMR was detected particularly against carbapenems, cephalosporins, fluoroquinolones, lincosamides, penicillins, and tetracyclines (Figure 4). No genetic determinants of AMR were described (Supplementary Data Sheet 2, Qualitative Analysis).

##### Enterococcus

The genus *Enterococcus* encompassed various public health and food relevant species such as *Enterococcus faecalis* and *Enterococcus faecium* as well as *Enterococcus avium* or *Enterococcus casseliflavus*. The bacteria originated from food of animal, vegetable and fruit origin. This included meat, milk, fish, seafood in raw, cut, shredded, whole, or fermented form. Soft cheeses but also semi-hard and hard cheeses were affected. These products originated from across Europe including Switzerland, but also from Argentina, Southeast Asia, and the USA. AMR was diverse and particularly predominant for aminoglycosides, fluoroquinolones, glycopeptides, lincosamides, macrolides, penicillins, and tetracyclines with lower presence also for carbapenems and cephalosporins (Figure 4, Supplementary Data Sheet 2, Qualitative Analysis).

##### Listeria

Among other species, *Listeria monocytogenes* was the most prominent member of this genus. Affected food products were of meat, seafood, fish, milk, vegetables and fruit origin. These included various types of cheeses, unprocessed, or fermented meat as well as raw vegetable or fruit preparations. The food products originated from various Southern European countries, but also Switzerland, Brazil, Canada, and the USA. AMR was detected predominantly against lincosamides and penicillins followed by aminoglycosides, fluoroquinolones, and cephalosporins (Figure 4). Genetic factors described were limited to *dfrA, dfrG*, and *tetM* (Supplementary Data Sheet 2, Qualitative Analysis).

##### Staphylococcus

The genus *Staphylococcus* was comprised of the species *S. aureus, S. epidermidis, S. carnosus, S. equorum*, and *S. saphrophyticus*. The food affected was mainly focused on animal derived products of meat and milk including raw, cut, ground, and fermented products as well as cheese. Relevant countries of product origin were mostly European countries including Switzerland. AMR was predominantly observed against penicillins, macrolides and tetracyclines followed by a lower prevalence of AMR against aminoglycosides, fluoroquinolones, glycopeptides and lincosamides (Figure 4). These AMRs were encoded for example by *aacA-aphD, aadD, blaI/R/Z, ermA/B/C/D/F/G/J/K, mecA, tetA/K/L/M*, or *vgaA/C* (Supplementary Data Sheet 2, Qualitative Analysis).

##### Technologically important bacteria

Technologically important bacteria were defined as those bacteria used directly to produce fermented food as well as probiotics. This includes particularly lactic acid bacteria, bifidobacteria isolated from food products, or starter cultures. The main species in this group belonged to genera *Bifidobacterium, Lactobacillus, Lactococcus, Leuconostoc, Pediococcus, Streptococcus*, and *Weissella*. The products were mainly of fermented meat, milk, and plant origin with the exception of strains directly isolated from starter or probiotic cultures or from unprocessed meat products. Countries of product origin included several European countries such as Switzerland but also Canada, Japan, and the USA. AMR was diverse and predominantly observed against aminoglycosides, fluoroquinolones, glycopeptides, penicillins, sulfonamides, and tetracyclines (Figure 4, Supplementary Data Sheet 2, Qualitative Analysis).

#### Other gram-positive and gram-negative bacteria

Bacteria not assigned to one of the above groups were classified as Gram-positive indicators (*Bacillus* spp.) or other Gram-positive and other Gram-negative bacteria. They were isolated from various raw meat, fish, and cheese products collected and produced in Europe including Switzerland as well as Canada and China (Supplementary Data Sheet 2, Qualitative Analysis). No quantitative assignment to samples was possible. AMR phenotypes were diverse and related for Gram-negatives particularly to aminoglycosides, cephalosporins, penicillins, and multiple other AM classes (Figure 4). Gram-positive organism mainly featured phenotypic AMR against aminoglycosides, lincosamides, macrolides, penicillins, phenicols, and tetracyclines (Figure 4). AMR genotypes were only described for Gram-negatives and included *bla*_CTX−M1_, *bla*_CTX−M14_, *bla*_OXA−48_ (Supplementary Data Sheet 2, Qualitative Analysis).

### Quantitative analysis by product categories

A total of 108 studies matched the inclusion criteria for quantitative analysis (Figure 1). The full dataset is available in Supplementary Data Sheet 3, Quantitative analysis.

#### Raw meat products

Raw meat products of beef, pork, poultry and game origin was primarily contaminated with *Campylobacter, E. coli, Enterococcus, Staphylococcus, Salmonella enterica* subsp. *enterica* serovars Typhimurium, Infantis and other serovars and to a lesser extent with *Yersinia enterocolitica* and *Listeria* (Supplementary Data Sheet 3, Quantitative Analysis). Median AMR prevalence was particularly high (>90%) for *Campylobacter* (cephalosporins, penicillins, tetracyclines, and fluoroquinolones from beef, pork, and mixed meat sources), *E. coli* (tetracyclines and trimethoprim from mixed meat sources), *Enterococcus* (streptogramins, nitrofurans, macrolides, lincosamides and tetracyclines from beef, pork, poultry and mixed meat sources), *Salmonella* (sulfonamides, penicillins and macrolides from pork, poultry, and mixed meat sources), *Staphylococcus* (streptogramins, pleuromutilins, macrolides from beef, poultry and veal sources) and *Yersinia* (macrolides from poultry sources) (Supplementary Data Sheet 3, Quantitative Analysis). *Enterococcus* and *Campylobacter* shared the highest diversity of 17 and 11 different AM classes with a median AMR prevalence >50%, respectively.

The exposure assessment showed highest exposure to AMRB (i.e., AMRB exposure score of 2) for *Campylobacter, E. coli, Enterococcus, Salmonella enterica* subsp. *enterica* serovars, and other non-*Enterococcus*/non-*Staphylococcus* indicator bacteria in meat of poultry and mixed animal sources (Supplementary Data Sheet 3, Quantitative Analysis).

Intermediate exposure to AMRB (i.e., AMRB exposure score of 1) was observed for pork, poultry and beef contaminated with *Campylobacter, Salmonella* serovars Enteriditis, Typhimurium, and other serovars as well as, *E. coli, Staphylococcus* and *Enterococcus* (Supplementary Data Sheet 3, Quantitative Analysis). While lamb meat displayed only low exposure scores, game meat had an intermediate exposure score for *Enterococcus*. The data was derived from a total of 164 studies, with higher level of evidence for poultry (*n* = 88), pork (*n* = 29), beef (*n* = 25) and mixed meat (*n* = 13) and particularly *Salmonella* spp. (*n* = 51), *E. coli* (*n* = 39), *Staphylococcus* spp. (*n* = 20), *Campylobacter* spp. (*n* = 18) and *Enterococcus* spp. (*n* = 15) whereas other sources or bacteria groups featured lower levels of evidence (Supplementary Data Sheet 3, Quantitative Analysis).

#### Fish and seafood

Fish and seafood products featured a median AMR prevalence (50–94%) for *E. coli* (glycopeptides and sulfonamides combinations), *Salmonella enterica* subsp. *enterica* (sulfonamides), *Staphylococcus* (aminoglycosides and phenicols), and *Vibrio* (penicillins and polypeptides) (Supplementary Data Sheet 3, Quantitative Analysis). Median AMR prevalence of 94% was only observed for *E. coli* (glycopeptides) and *Vibro* (polypeptides). AMR exposure score reached a maximum of 1 for *Vibrio, E. coli* and *Enterococcus* (Supplementary Data Sheet 3, Quantitative Analysis). Level of evidence was supported by 47 studies.

#### Plant-based food

Plant-based food products, mainly raw or mechanically treated vegetables and fruits, were mostly contaminated with *E. coli* (lincosamides, nitrofurans, and rifamycins), *Salmonella* (nitrofurans) and other indicator organisms (penicillins and trimethoprim) with a median AMR prevalence of 57–64%. Only *E. coli* (lincosamides) featured a median AMR prevalence of 91% (Supplementary Data Sheet 3, Quantitative Analysis). The AMR exposure score reached a maximum level of 1 for *S. enterica* of various serovars, *Listeria, E. coli, Enterococcus* and other indicator bacteria (Supplementary Data Sheet 3, Quantitative Analysis). The level of evidence was supported by 25 studies.

#### Raw milk and cheese

Raw milk featured highest median AMR prevalence of 57% for *Staphylococcus* (penicillins). Cheese showed highest median AMR prevalence of 63 and 98% against aminoglycosides and fluoroquinolones among the technologically important bacteria including starter cultures (Supplementary Data Sheet 3, Quantitative Analysis). Besides, high AMR exposure score of 2 was observed in cheese for technologically important bacteria including starter cultures and a level of 1 for *Enterococcus* Raw milk only featured low exposure score for the bacteria included in this study (Supplementary Data Sheet 3, Quantitative Analysis). The level of evidence was supported by 9 studies.

### Risk of bias

Out of the 1,065 studies retained for full screening and data extraction, 137 had to be excluded from further analysis because of poor design quality or poor reporting of research findings, which made it impossible to identify the sample or isolate source (Figure 1). Out of the 313 studies included into this evaluation, 108 qualified for the strict sample and isolate requirements for quantitative analysis. A total of 208 were analyzed only on a descriptive level due to not being classified as regular prevalence study (*n* = 119), inability to differentiate food categories (*n* = 2), insufficient bacteria identification (*n* = 3), insufficient food samples (*n* = 23), insufficient bacteria prevalence (*n* = 21) or AMR prevalence (*n* = 7) data or insufficient isolate numbers (*n* = 34) (Figure 1).

Based on our three point scale to rate the studies in relation to their sampling scheme, 263, 34, and 17 studies out of 313 were assigned to (1) convenient sampling, (2) systematic approach, not further specified, and (3) systematic weighted and probabilistic sampling, respectively (Supplementary Data Sheet 1). This suggests that the majority of studies has a high potential risk of bias and the available knowledge from systematic probabilistic prevalence studies is limited to 17 studies. Among the 108 studies used for quantitative analysis, the risk of bias was reduced with a distribution of 74, 20, and 14 for the bias categories (1), (2), and (3), respectively (Supplementary Data Sheet 1).

## Discussion

### General aspects of AMR exposure via food

In this systematic literature review, we aimed to estimate the AMR exposure of consumers based on food sold at retail on the example of Switzerland. The study is positioned to provide literature-based estimations for AMRB prevalence and exposure data in food at retail. The AMR exposure estimations form a basis for the design of an appropriate follow-up AMR risk assessment at retail and consumer level integrated into systematic surveillance systems.

Switzerland was selected for its strong local agriculture production in parallel to the large and well-documented import of food products from nearly all regions of the world to obtain a more global relevance for this study. As demonstrated for vertical transfer via breast milk from mother to the gastrointestinal tract (GIT) of infants (Jost et al., 2014), bacteria can transfer from food to the GIT microbiota. AMR gene transfer can then occur in the favorable conditions of the GIT between bacteria members of the GIT microbiota (Haug et al., 2011) and thus present a public health risk.

Quantifiable AMR exposure was particularly high for meat products in relation with foodborne pathogens such as *Campylobacter* and *S. enterica* subsp. *enterica*, but also indicator organisms of *E. coli* and *Enterococcus* covering AMR particularly to highest priority critically important AM such as cephalosporins, fluoroquinolones and macrolides (World Health Organization, 2016). AMR surveillance reports in Switzerland and the European Union regarding zoonotic pathogens in pre-slaughter livestock poultry, pig and cattle report ampicillin, sulfonamides, and tetracycline resistances in *Salmonella* as well as ciprofloxacin and tetracycline in *Campylobacter* as highly prevalent AMRs (EFSA and ECDC, 2016; Federal Office of Public Health Federal Food Safety Veterinary Office, 2016). In this study, the major AMRs among Gram-negative foodborne pathogens were observed in *Campylobacter* against fluoroquinolones and tetracyclines. In *Salmonella*, main phenotypic AMR was detected against aminoglycosides, cephalosporins, fluoroquinolones, penicillins, sulfonamides, and tetracyclines with high dependency of the AMR profiles on serovars. *Campylobacter*, although at lower prevalence, featured also macrolide resistances, which correlate with the increasing concerns regarding macrolide AMR presence and transferability (Wang et al., 2014; EFSA and ECDC, 2016). Indicator bacteria *E. coli* featured AMR to aminoglycosides, penicillins, fluoroquinolones, sulfonamides and tetracyclines. *Enterococcus* displayed AMR to a multitude of AM classes including aminoglycosides, fluoroquinolones, glycopeptides, macrolides, penicillins, and tetracyclines. Systematic literature review data of food at retail therefore match the key AMRs as reported for Europe (EFSA and ECDC, 2016; Federal Office of Public Health Federal Food Safety Veterinary Office, 2016). However, existing reports and datasets focused mainly on Gram-negative and Gram-positive foodborne pathogens and indicator organisms in meat products (EFSA and ECDC, 2016). Consequently, only limited knowledge is available regarding AMRB in other food categories as well as AMR in technologically important bacteria including starter cultures. Therefore, this study provides a systematic overview and estimation of AMR exposure data for food at retail covering a broad spectrum of food categories and bacteria groups as a prerequisite for the development of a comprehensive AMR risk assessment for the consumer.

The risk for consumers is multifactorial and depends also on food preparation and consumption habits. As most raw meat products undergo a cooking step prior to consumption that reduces the bacteria quantity, the final AMRB exposure level can largely vary depending on hygiene practices (Røssvoll et al., 2015). For campylobacteriosis and non-typhoidal salmonellosis as predominating zoonotic foodborne infections, 96 and 79 million infection cases are reported worldwide each year, respectively (Havelaar et al., 2015). Switzerland as study area reported over 7,500 confirmed campylobacteriosis cases in 2014 to yield an incidence rate of 93 cases per 100,000 persons, over 70% of which were related to poultry origin (BLV, 2015). This is particularly linked to chicken meat fondue dishes in Switzerland and other European countries where shared plates or cutlery for raw and cooked meat lead to cross-contamination from raw chicken to cooked food. The consumption of raw chicken meat in meat fondue represents a key risk factor for the annual campylobacteriosis winter peak (Bless et al., 2014). This suggests that appropriate hygiene practices are not systematically applied (Macdonald et al., 2015). Raw meat products therefore represent a major factor for cross-contamination of bacteria in the kitchen or at the table to infect humans and therefore likely also for the transfer of AMRB to humans.

In contrast to raw meat or other food products undergoing a cooking step, fermented products are generally consumed without a prior heating step and feature a high level of 10^8^-10^9^ colony counts of technologically relevant bacteria and indicator bacteria per gram of product (Ross et al., 2002). Therefore, AMRB if present might transfer in significantly higher numbers from food directly to the consumer. Particularly members of the technologically important bacteria and *Enterococcus* in cheese featured highest and intermediate AMR exposure scores, respectively. As limited data hindered further exposure calculations for other fermented products as well as utilized starter cultures, qualitative analysis in this study indicated that AMRB in fermented food products, starter cultures, and *Enterococcus* are present and require enhanced systematic surveillance. This surveillance scheme should include the determination of the potential for AMR gene transfer between starter culture bacteria e.g., lactic acid bacteria, as well as to gut commensals, opportunistic, and obligate pathogens in order to estimate the associated public health risk.

The AMR risk assessment is related to phenotypic and genotypic AMR determination in foodborne pathogens and indicator bacteria as well as the potential dissemination of these AMR genes to other pathogens. AMR genotypes allow elucidating the epidemiology of certain AMR traits in different environments (Aminov and Mackie, 2007; Logan and Weinstein, 2017). The genetic repertoire of standard foodborne pathogens and indicator bacteria were well investigated in the studies included for this review. However, data on AMR genetics is still limited for public health relevant bacteria that are not as frequently isolated from food such as *C. difficile*. Furthermore, quantifiable data on AMR gene prevalence as well as the detailed elucidation of genetic linkages between AMR genes and mobile genetic elements such as plasmids, transposons or integrons is rarely reported (Lanza et al., 2015; Martínez et al., 2017). This unfortunately hinders better estimations on AMR gene prevalence and transferability. This is particularly relevant in relation to the transferability of AMR genes located on chromosomes vs. those on mobile genetic elements in terms of clonal expansion vs. horizontal transfer as exemplified for *Campylobacter* spp. and *Enterobacteriaceae* (EFSA and ECDC, 2016). AMR genes in these organisms previously considered as not horizontally transferrable such as those encoding for colistin and erythromycin resistances were observed in horizontal gene transfer events in *Enterobacteriaceae* and *Campylobacter* respectively (Wang et al., 2014; EFSA and ECDC, 2016; Florez-Cuadrado et al., 2016). On the example of *Campylobacter* horizontal gene transfer of erythromycin resistance was associated with *ermB* being located on a chromosomal multidrug resistance genomic islands, which rendered all recipients also resistant to lincosamides and aminoglycosides (Wang et al., 2014). Therefore, the study of the genetic organization of AMR genes is of high importance for an AMR risk assessment and should be more systematically implemented.

### Discussion of AMR prevalence in Switzerland and swiss food products

Switzerland as selected study location contributed only a small amount of locally produced (0.5%) and locally collected (2%) food samples to the total set of samples included. However, the prevalence of AMRB in samples originating from food produced and collected in Switzerland (49.1 and 35.7%, respectively) was significantly higher than the average of 24.6% (Table 1). These differences between Switzerland and the other geographic areas were less prominent in comparison to the overall set of European samples featuring 29.8% AMR prevalence. Besides, the AMR strain prevalence of 33.0% in Switzerland was well within the data range of 16.0–38.5% in the other regions. Evaluated by AMR prevalence by food categories such as meat (cuts, ground, pieces, meatballs), meat not further specified and cheese, values for Swiss samples of 37.6, 27.6, and 19.1% were comparable to those of global origin of 35.5, 32.6, and 26.2%, respectively (Tables 3, 4). This suggests that the consumer exposure to AMRB in Switzerland is likely comparable to other places worldwide. This finding also supports the expansion of our systematic literature review to data from countries representing the main sources of food import to Switzerland in order to obtain additional AMRB exposure estimations. This does by no means imply a direct possible generalization of AMR prevalence findings among samples analyzed globally to the products on the Swiss market. However, given the AM pressure and AMR prevalence in the production areas of food imported and that in domestic production as well as the fact that global food and livestock trade can contribute to the dissemination of AMRB (Mo et al., 2014), systematic surveillance of retail food of any origin should be implemented to provide an accurate AMRB risk assessment at retail and for the consumer.

### Identified knowledge gaps, limitations, risk of bias, and future recommendations

The initial search retrieved a significant number of >16,000 references to be reduced to 1,065 after initial curation and yielding a final 313 studies qualified for data extraction according to the defined inclusion and exclusion criteria. Unfortunately, 137 out of 1,065 studies of interest had to be excluded due to insufficient differentiation between sources of isolates, inability to extract data from the provided data or general quality concerns.

The final dataset (*n* = 313) retrieved for this study featured a potentially high risk of bias due to the limited availability of systematic probabilistic AMR prevalence studies (*n* = 17) of food at retail level. Within the 108 studies qualified for quantitative analysis, the relative abundance of systematic probabilistic AMR prevalence studies was increased. In the absence of sufficient number of studies with low risk of bias, studies based on convenient sampling schemes still provided useful data to obtain an initial AMR exposure estimation. This clearly highlighted a significant knowledge gap relating to systematic analysis of AMR prevalence in food at retail level.

A further bias was observed related to food category types. Meat products represented 63.5% of all samples (Table 3). This bias originated from 164 and 47 studies available for meat and seafood products, whereas only 25 and 9 studies investigated plant and dairy products, respectively. Out of these, a mere seven studies reported on fermented products. Similarly, novel food such as insects were rarely assessed for the prevalence of AMRB and will also require increased surveillance activities due to growing interest, different consumption habits, and utilization as animal feed (Van Huis, 2013; Stamer, 2015).

Similar bias were observed for geography and reporting of production origin. Europe and North America were responsible for 30.0 and 59.5% of all samples analyzed, respectively, whereas information on the country of production was absent in nearly 80% of samples (Table 3). In terms of global exposure to AMRB via food, a more comprehensive documentation of the occurrence of AMR throughout food supply chains would be favorable. In combination with the issue around small sample sizes, this review highlighted the need for standardized surveillance schemes of food at retail. This would provide improved data for meta-analysis and enhance statistical power for AMRB exposure calculations. Besides, more complete data for AMR surveillance at healthcare, farm level, food/consumer level and in the environment is warranted for a truly overarching One Health approach to AMR.

A potential bias was observed for the targeted bacteria species. *E. coli* (*n* = 31,838), *Salmonella* (all serotypes, *n* = 13,547), *Enterococcus* (*n* = 13,502), and *Campylobacter* (*n* = 10,269) were responsible for the major share of observed AMR phenotypes (Figure 4). This was due to *Salmonella, E. coli, Staphylococcus, Enterococcus*, and *Campylobacter* being represented by 74, 56, 30, 19, and 19 studies, respectively. These bacteria also yielded the highest AMRB exposure scores while most other food categories or bacteria species had to be excluded due to insufficient sample sizes for quantitative analysis. This was particularly observed for technologically relevant bacteria investigated in only two studies included for quantitative analysis and suggesting a further knowledge gap.

On a descriptive analysis basis, these potential knowledge gaps were supported by indications for AMRB exposure via food categories with insufficient datasets. This finding was particularly significant for fermented products (meat, milk, and plant origin), dairy products in general, veal and game meat, novel food (insects), probiotics and technologically relevant bacteria species (including starter cultures). Descriptive analysis of these categories suggested high levels of AMR. Systematic surveillance data retrieved in this systematic review is insufficient to properly assess the risk of exposure through these products. However, these food categories products should be considered by no means as less relevant for consumer AMRB exposure, particularly due the consumption patterns often without prior heat treatment of fermented food products and thus potential transfer of AMR genes in the GIT. The genetic background of these AMRs, particularly the involved genes and mobile genetic elements, could only be assessed on a descriptive basis but not quantitatively due to the means of data reporting from the included studies and often complete absence of information on genetic location of the AMR genes.

## Conclusions

This study elaborates the level of human exposure to AMRB via food at retail including the identification of several surveillance and knowledge gaps in relation to AMRB in food. High levels of AMRB exposure were observed for raw meat, fish and plant products regarding Gram-negative *Campylobacter, E. coli, Salmonella, Vibrio*, and *Yersinia* with AMR against aminoglycosides, cephalosporins, fluoroquinolones, penicillins, sulfonamides, and tetracyclines and Gram-positive *Enterococcus, Listeria*, and *Staphylococcus* with AMR against aminoglycosides, fluoroquinolones, glycopeptides, lincosamides, macrolides, nitrofurans, penicillins, sulfonamides, streptogramins, and tetracyclines. For raw meat and fish products, the exposure to these AMRB is high. However, the risk of AMRB transmission to humans can likely be reduced using adequate food processing techniques as well as high hygiene standards in the kitchen and at the table to avoid cross-contamination.

In addition, fermented dairy products featured high AMRB exposure regarding *Enterococcus* and technologically important bacteria such as starter cultures with AMR against aminoglycosides, fluoroquinolones, glycopeptides, penicillins, sulfonamides, and tetracyclines. Particularly *Enterococcus* featured a broad range of resistances toward AM classes of Gram-positive and Gram-negative relevance. Accurate exposure quantification was however limited by low availability of AMR prevalence data. In contrast to raw meat, ready-to-eat-products such as plant- or fermented products are associated with a high transmission risk through the absence of a cooking step prior to consumption. This suggests a potentially underestimated role of fermented foods and technologically important bacteria in AMRB exposure and therefore a major knowledge gap.

Other observed knowledge gaps relate to the genetic organization of AMR genes in mobile genetic elements and multidrug resistance genomic islands. In addition, the potential risk of bias involved with AMR exposure estimations based on systematic literature review data limit the ability to provide a direct risk assessment for the consumer.

Nevertheless, this review provided pivotal information on AMRB exposure in food at retail for the design of future AMR risk assessment studies based on actual Swiss data and will help to optimize AMR surveillance schemes. This particularly concerns (i) the implementation of systematic AMR surveillance schemes of food at retail, (ii) the inclusion of additional food categories such as ready-to-eat, plant, fermented food, and novel food products, (iii) the addition of further bacteria groups such as technologically important bacteria, and (iv) the investigation of AMR genetics. We thus recommend to design and enforce systematic phenotypic and genotypic surveillance of AMR in retail food as an additional core pillar of a One Health strategy for AMR surveillance and response systems.

## Author contributions

CJ and ES developed the literature data search strings, performed the searches and data extractions. LC performed quantitative data analysis. KDCS, LM, and RS supervised the work. CJ wrote the first manuscript draft. All authors contributed to data analysis, interpretation and provided substantial input to the manuscript. All authors have read and approved the submitted version of the manuscript. All authors agree to be accountable for all aspects of the work in ensuring that questions related to the accuracy or integrity of any part of the work are appropriately investigated and resolved.

### Conflict of interest statement

KDCS and LC were employed by company SAFOSO AG, Switzerland. The other authors declare that the research was conducted in the absence of any commercial or financial relationships that could be construed as a potential conflict of interest.
